# Large-Scale and Site-Specific Mapping of the Murine Brain *O*-Glycoproteome with IMPa

**DOI:** 10.1021/acs.analchem.3c00408

**Published:** 2023-08-25

**Authors:** Suttipong Suttapitugsakul, Yasuyuki Matsumoto, Rajindra P. Aryal, Richard D. Cummings

**Affiliations:** Department of Surgery, Beth Israel Deaconess Medical Center, Harvard Medical School, Boston, Massachusetts 02215, United States

## Abstract

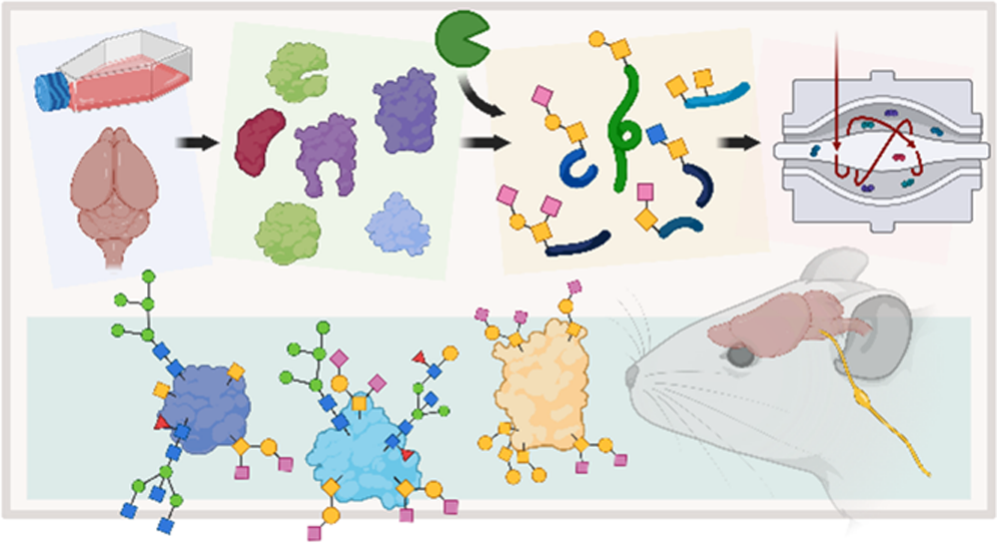

Altered protein glycosylation is typically associated with cognitive defects and other phenotypes, but there is a lack of knowledge about the brain glycoproteome. Here, we used the newly available *O*-glycoprotease IMPa from *Pseudomonas aeruginosa* for comprehensive *O*-glycoproteomic analyses of the mouse brain. In this approach, total tryptic glycopeptides were prepared, extracted, purified, and conjugated to a solid support before an enzymatic cleavage by IMPa. *O*-glycopeptides were analyzed by electron-transfer/higher-energy collision dissociation (EThcD), which permits site-specific and global analysis of all types of *O*-glycans. We developed two complementary approaches for the analysis of the total *O*-glycoproteome using HEK293 cells and derivatives. The results demonstrated that IMPa and EThcD facilitate the confident localization of *O*-glycans on glycopeptides. We then applied these approaches to characterize the *O*-glycoproteome of the mouse brain, which revealed the high frequency of various sialylated *O*-glycans along with the unusual presence of the Tn antigen. Unexpectedly, the results demonstrated that glycoproteins in the brain *O*-glycoproteome only partly overlap with those reported for the brain *N*-glycoproteome. These approaches will aid in identifying the novel *O*-glycoproteomes of different cells and tissues and foster clinical and translational insights into the functions of protein *O*-glycosylation in the brain and other organs.

## Introduction

Glycosylation plays important roles in cells by affecting protein functions, properties, interactions, and activities.^1−3^ Aberrant protein glycosylation as identified in congenital disorders of glycosylation (CDG) is often associated with altered neurological functions, cancer, auto-immunity, and chronic inflammation.^4−6^ Tumor cells express altered *O*-glycans including the Tn and sialyl Tn antigens,^7−9^ IgA nephropathy is associated with altered *O*-glycosylation of IgA1,^10,11^ and Tn syndrome is an acquired auto-immune disease caused by a missing galactose residue from the T antigen, leaving the Tn antigen present on blood cells instead.^12^ More importantly, changes in protein glycosylation at specific sites have also been implicated in several disorders and conditions in human diseases.^13−16^ It is obviously imperative to investigate site-specific *O*-glycosylation patterns on glycoproteins in healthy and diseased tissues. Mass spectrometry (MS)-based glycoproteomics is currently the best approach to identify protein glycosylation on a large scale and in a site-specific manner, especially with current technologies that allow for intact glycoproteomics analysis without the need to deglycosylate, simplify, or modify the glycans.^17−19^ These can improve our knowledge of protein glycosylation in biological processes and could potentially lead to the discovery of drug targets or biomarkers for disease detection.

Several studies have reported on the *N*-glycoproteome;^20−23^ however, the study of the *O*-glycoproteome for the brain or other organs is not yet broadly practical due to many factors.^24^ The localization of *O*-glycosylation sites is difficult, as *O*-glycans are often clustered together within a domain, which impacts proteolytic cleavage by commonly used enzymes in proteomics analysis.^25,26^ Tryptic *N*-glycopeptides mostly contain one glycosylation site with a consensus sequence, making the glycosylation site localization more predictable,^20^ yet there is no consensus site for *O*-glycosylation. Unlike PNGaseF used in *N*-glycoproteomics analysis to remove N-glycans, there is no common enzyme to deglycosylate *O*-glycans and generate a common tag for site determination. To overcome these difficulties, many different approaches for MS-based *O*-glycoproteomics have been explored. The SimpleCell (*Cosmc*KO) technology was developed for *O*-glycosylation site identification,^27^ in which cells are engineered to simplify *O*-glycans expression to the Tn antigen, and lectins, such as VVA, that bind this antigen are used for *O*-glycopeptide enrichment. The method termed IsoTaG is used for intact *N*- and *O*-glycopeptide analysis, which involves metabolic labeling of *O*-glycopeptides with a sugar analogue and tagging with a cleavable probe for targeted glycoproteomics analysis.^28^ Unfortunately, the metabolic labeling step is not compatible with most clinical or biological samples. Yang and colleagues introduced a method for site-specific extraction of *O*-linked glycopeptides (EXoO), as documented for glycoproteins from human kidney, serum, and T-cell samples.^29,30^ OgpA employed in that method cleaves N-terminal to glycosylation sites with mainly the non-sialylated core 1 *O*-glycan,^29,31^ as glycans must be desialylated for the full enzymatic activity of OgpA. Hence, information on *O*-glycan sialylation and other features is lost. Several mucin *O*-glycoproteases have also been reported for *O*-glycoproteomics;^32−34^ however, these mainly showed activities toward highly glycosylated mucin domain.^31,32^

Recently, a novel immunomodulating metalloprotease (IMPa) from *Pseudomonas aeruginosa* was identified that can cleave *O*-glycopeptides.^31,35,36^ The enzyme efficiently cleaves N-terminal to a serine or threonine residue containing an *O*-glycan, including core 1 *O*-glycans, sialylated core 1 *O*-glycans, the Tn antigen, and other *O*-glycans with varying complexity. While this approach has been used for site-specific analysis of several glycoproteins, global analysis of *O*-glycoproteins in cells and tissues has not been performed with IMPa.

Here, we used IMPa to globally and site-specifically identify *O*-glycoproteins and glycosylation sites from the murine brain and several cultured cell types by employing IMPa. Glycopeptides from biological samples were first conjugated to a solid support, and IMPa was used to cleave *O*-glycopeptides for intact glycoproteomics analysis. We tested the approach using both wild-type and SimpleCell HEK293 cells and identified various site-specific *O*-glycans including those that are sialylated and the Tn antigen. The use of EThcD fragmentation further resolved and improved the localization of *O*-glycans on the glycopeptides. We coupled this approach with 2D fractionation to investigate the *O*-glycoproteome of mouse brains and showed that the majority of the glycoproteins contain di-sialylated core 1 *O*-glycans. This new approach dramatically expedites the analyses of *O*-glycoproteomes in different biological samples.

## Experimental Section

### Cell Lysis, Protein Extraction, and Peptide Purification

Extended experimental details can be found in the Supporting Information. HEK293 cells or mouse brain tissues were lysed. The resulting proteins were reduced, alkylated, and digested with sequencing-grade modified trypsin (Promega) overnight.

### Enzymatic Release of *O*-Glycopeptides with IMPa

Methods for glycopeptide extraction and immobilization were adapted and modified from Yang et al.^29,30^ Briefly, peptides were desalted with C18 Sep-Pak Vac cartridges (Waters), guanidinated, and purified. For total *O*-glycoprotein experiments, the glycopeptides were enriched with HyperSep Retain AX Cartridges (RAX, Thermo). For Tn-focused experiments, the glycopeptides were enriched with agarose-bound VVA beads (Vector). The eluted glycopeptides were purified and conjugated to AminoLink Plus Coupling Resin (Thermo). *O*-glycopeptides were released by treating with IMPa *O*-glycoprotease (New England BioLabs) overnight. The eluted glycopeptides were collected and purified. For the mouse brain experiment, the dried peptides were fractionated with high-pH reversed-phase HPLC.

### LC-MS/MS Analysis, Database Searching, and Bioinformatics Analysis

Peptide sequencing was performed on a Dionex UltiMate 3000 UHPLC system coupled to an Orbitrap Fusion Lumos Tribrid mass spectrometer (Thermo). Higher-energy dissociation product ion-triggered electron-transfer/higher-energy collision dissociation (HCD-pd-EThcD MS2) or HCD MS2 alone was used for glycopeptide identification.^37−39^ Raw files were searched using pGlyco3^40^ against the human or mouse proteome database. The glycopeptide false discovery rate (FDR) was 1%. Identified glycopeptides were inspected and filtered manually.

## Results and Discussion

### Identification of *O*-Glycoproteins from HEK293 Cells with IMPa

**Figure 1:**
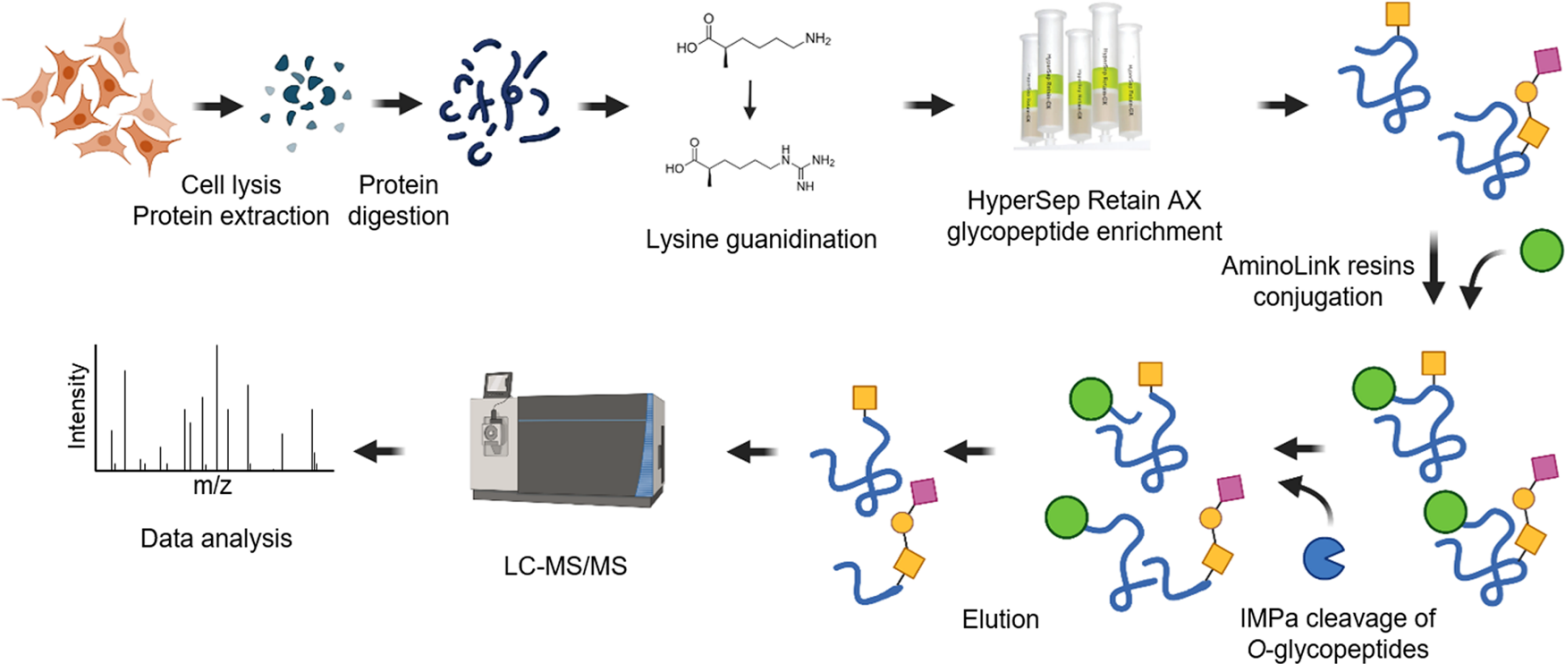
Experimental scheme. Cells or tissues are first lysed. The proteins are extracted, digested, guanidinated, and purified. Glycopeptides are enriched with HyperSep Retain AX and conjugated with AminoLink resins through a reductive amination reaction. The immobilized *O*-glycopeptides are cleaved with IMPa and purified. Eventually, *O*-glycopeptides are analyzed with LC-MS/MS for glycopeptide identification and site localization.

Among the novel *O*-glycoproteases recently reported in the literature, the *O*-glycoprotease named IMPa from *P. aeruginosa* is particularly interesting.^31^ Compared to OgpA, IMPa is superior as it can recognize and cleave glycopeptides with various *O*-glycans, including those that are sialylated. We first aimed to test the capability of IMPa for general *O*-glycoproteomics analysis and used an experimental scheme where IMPa is employed for the cleavage of *O*-glycopeptides (Figure 1).^29,30^ We used both wild-type HEK293 and the Tn-expressing SimpleCell HEK293 cells for the analyses of *O*-glycans and those with the Tn antigen. Here, the glycopeptides were enriched with RAX columns and conjugated to AminoLink resins through a reductive amination reaction. *O*-glycopeptides were eventually released using IMPa treatment and subsequently analyzed with LC-MS/MS.

With this approach, we identified a total of 123 protein groups with 25 *O*-glycan compositions from 1030 glycopeptide-spectrum matches (GSMs) from a single-shot analysis and higher-energy collision dissociation (HCD) fragmentation (Supporting Information). Among these, 100 protein groups and 23 glycan compositions were identified from 980 GSMs with serine or threonine at the peptide N-terminus, which is consistent with the experimental design where IMPa should cleave N-terminal to the *O*-glycosylation site. Thirty-nine glycoprotein groups from 286 glycopeptides contained a HexNAc(1) glycan with serine or threonine at the N-terminus, indicating the identification of Tn glycoproteins with GalNAc-Ser/Thr, as IMPa only cleaves *O*-GalNAcylated, but not *O*-GlcNAcylated glycopeptides. Several glycopeptides with sialylated *O*-glycans, including 53 glycoprotein groups from 378 glycopeptides with one and two sialic acid residues on core 1 *O*-glycan, were also identified. Only 8 glycoprotein groups contained the non-sialylated core 1 *O*-glycans. With EXoO, where glycopeptides are treated with both sialidase and OgpA, these 53 glycoprotein groups would be identified as having core 1 *O*-glycan. Hence, our analysis with IMPa further improved the glycosylation information from the current technology.

Compared with other reported methods, we expected that this approach would yield a lower coverage due to (1) the single-shot analysis of *O*-glycopeptides used in the current section and (2) the native *O*-glycan state on various glycoproteins. With the SimpleCell approach, 259 glycoproteins were identified from the total cell lysate and secretome of HEK293 cells.^27^ In that study, the peptides were fractionated into 12 samples prior to MS analysis, which increased the chance of peptides being detected by mass spectrometry. SimpleCell *O*-glycopeptides contained the Tn antigen, which decreased the glycopeptide heterogeneity compared to the native state and greatly improved the abundance of glycopeptides that can be detected since they are more homogeneous. It should also be noted that the study was published in 2013, and the instrument was not as sensitive. In this initial experiment, we also employed HCD for glycopeptide identification. With the EXoO method, the authors noted that some glycans, such as HexNAc(2)Hex(2), might not be resolved and could result from two HexNAc(1)Hex(1) glycans on a single peptide.^29,41^ This was also observed in our experiments and previously.^41^ To overcome this problem, we used EThcD as described below.

Of note, with several trial experiments, we observed that the optimal starting protein amount was ∼40 mg from the lysates. In a separate experiment where the starting protein amount was decreased by half, we also observed a 50% decrease in glycoprotein coverage. We also noted that RAX was based on SAX-ERLIC separation mode, and thus, the charge or hydrophilicity of the glycopeptides might affect the retention of some *O*-glycopeptides, especially for the hydrophilicity since the glycopeptides were trapped in 95% ACN with 1% TFA where the column behaves in the HILIC mode. Comparison of enzymatic cleavages by OgpA and IMPa, and even with newer *O*-glycoproteases that have been recently discovered such as SmE,^34^ on a large scale could also reveal the substrate preference for these *O*-glycoproteases. Yet, the limiting factor that may arise is the large protein amount required in our method.

### Identification of the Tn Glycoproteome from SimpleCell HEK293 Cells

The Tn antigen is an important *O*-glycosylation motif involved in critical biological processes such as protein folding and expressed in disease states, such as cancer.^42−44^ Previously, Yang and colleagues also modified the EXoO method to target the Tn glycoproteome.^45^ In that approach, Tn glycopeptides were first enriched with the lectin VVA that binds the Tn antigen. The enzyme T-synthase (C1GalT1) was then employed to transfer ^13^C_6_-Gal from UDP-^13^C_6_-Gal to Tn glycopeptides, which were then cleaved by OgpA. This generates core 1 *O*-glycopeptides with a heavy tag on Gal that can be distinguished from the *de novo* core 1 *O*-glycan by MS analysis. The success of this approach relied on the reaction efficiency of the transfer reaction by T-synthase. To improve the specificity in our experiment for the Tn antigen identification and to skip the reaction by T-synthase, we replaced RAX enrichment with VVA enrichment. Since IMPa can cleave Tn glycopeptides directly, there was no need to enzymatically label the peptides to distinguish *de novo* core 1 *O*-glycan with the newly generated, isotopically labeled ones. Here, Tn glycopeptides were enriched by an overnight incubation with VVA agarose beads. After peptide purification, the glycopeptides were conjugated to a solid support and treated with IMPa similarly.

We observed that HCD fragmentation alone could not localize the *O*-glycosites efficiently, as supported by other previous studies.^29,41,46^ We employed EThcD for *O*-glycoproteomics analysis and checked whether VVA enrichment alone was sufficient for Tn glycoproteomics analysis, specifically with the higher-energy dissociation product ion-triggered electron-transfer/higher-energy collision dissociation (HCD-pd-EThcD) approach where oxonium ions in scout HCD scans that indicate the presence of glycopeptides trigger the subsequent EThcD acquisition.^46^ Overall, HCD fragmentation resulted in a higher coverage of proteins identified than those with HCD-pd-EThcD acquisition from both VVA enrichment only and VVA with the subsequent IMPa reaction, possibly due to the faster cycle time of the HCD-only acquisition (Supporting Information). However, this was not sufficient for glycosylation site localization. With HCD-pd-EThcD experiments, the coverage was higher in the experiment with VVA followed by the subsequent IMPa reaction. The coverage from the VVA followed by IMPa was also generally higher than the VVA enrichment only, which may be due to the more enriched Tn glycopeptides in the samples. We also compared the localization score for those identified by EThcD (Figure S1). The average localization score for the VVA+IMPa experiment was higher than the VVA alone experiment (22 vs 18, respectively). In the VVA+IMPa experiment with HCD-pd-EThcD fragmentation, we identified a total of 97 protein groups that contained the Tn antigen on 210 unique glycosylation sites and from 583 GSMs from a single-shot analysis. Therefore, we concluded that VVA enrichment with IMPa reaction and EThcD fragmentation was necessary for *O*-glycoproteomics analysis, especially for the Tn glycoproteome.

### Large-Scale Identification of Mouse Brain *O*-Glycoproteome

**Figure 2:**
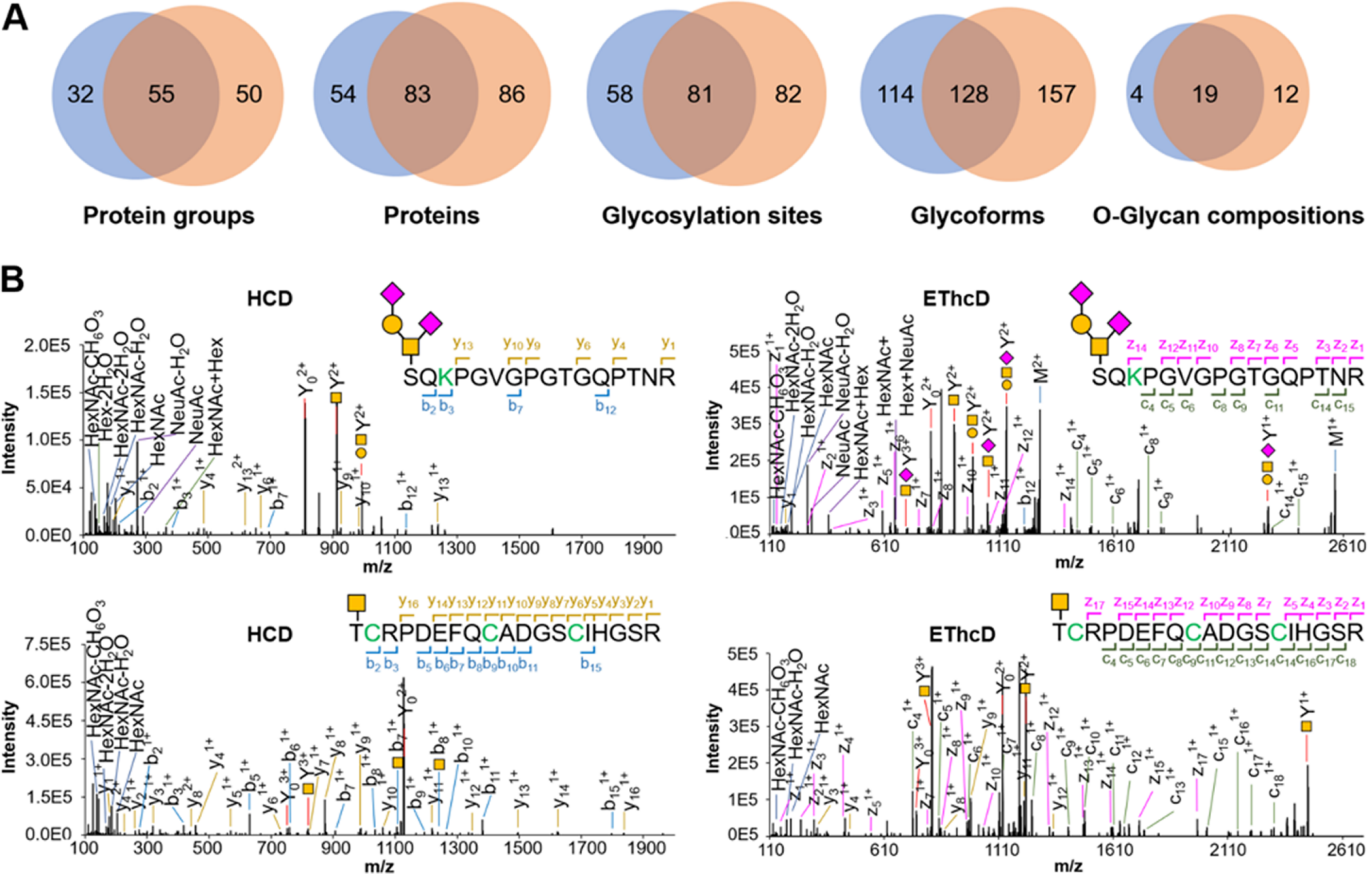
Identification of *O*-glycoproteins from mouse brains. (A) Overlaps of protein groups, proteins, glycosylation sites, glycoforms, and glycan compositions from two biological replicate experiments in mouse brains. Note that biological replicate two was from three technical replicates. (B) Example MS2 spectra of *O*-glycopeptides detected from the mouse brain. (Top) HCD and EThcD spectra of S[HexNAc(1)Hex(1)NeuAc(2)]QKPGVGPGTGQPTNR from Cspg5. (bottom) HCD and EThcD spectra of T[HexNAc]CRPDEFQCADGSCIHGSR from Ldlr. Each HCD spectrum resulted in EThcD fragmentation of the precursor ions through EThcD. Amino acid residues in green are modified by carbamidomethylation (C) or guanidination (K).

Using these approaches with IMPa, we characterized the *O*-glycoproteome in mouse brains. Little is known about the *O*-glycosylation of brain glycoproteins, but multiple studies indicate that altered protein glycosylation is associated with neurological symptoms.^47^ We identified over 100 glycoprotein groups, 200 glycosylation sites, 300 glycoforms, and 35 *O*-glycan compositions from two biological replicates, single-shot experiments (Figure 2A). To maximize the coverage of *O*-glycopeptides, we additionally employed 2D fractionation in a high-pH reverse-phase mode after *O*-glycopeptides were released from the AminoLink resins (Figure S2). The fractions were collected, concatenated into 12 samples, and analyzed with LC-MS/MS. In total, including (a) single-shot experiments, (b) experiments where the peptide load was increased, and (c) experiments with a modified MS1 scan range, we detected 367 *O*-glycoprotein groups (613 individual possible *O*-glycoproteins), 80 *O*-glycan compositions, 576 *O*-glycosylation sites, 1431 *O*-glycoforms, and 695 unique *O*-glycopeptides (Supporting Information). Over 93% of the GSMs have the 138/144 ratio less than 3, indicating that these are *O*-glycopeptides (Figure S3, Supporting Information).^46^ Those with a higher ratio mostly contain a HexNAc(2)Hex(2) composition, which was predicted to be a core-2 *O*-glycan that has a GlcNAc residue. Tissue expression and gene ontology analysis showed that these identified proteins are from the brain and function corresponding to the general roles of glycoproteins (Figure S4, Supporting Information).

We compared the mouse brain *O*-glycoproteins with publicly available data sets for *N*-glycoproteins, including intact *N*-glycoproteins from normal human brains,^48^ deglycosylated *N*-glycoproteins from mouse brains of the same strain,^22^ and intact *N*-glycoproteins from mouse brains of the same strain.^17^ Unexpectedly, the overlap between identified *N*- and *O*-glycoproteins was minimal in each comparison (Figure S5). We used NetNGlyc to predict N-glycosylation on the identified *O*-glycoproteins.^49^ Among the 613 total *O*-glycoproteins, 592 were determined to have a consensus sequence for N-glycosylation (N-X-S/T, where X can be any amino acid residues except proline. Proteins with >4000 amino acid residues were omitted since NetNGlyc cannot analyze these proteins). Among these, 586 *O*-glycoproteins (98.99%) were predicted to have at least one *N*-glycosylation site even though the majority of them were not experimentally identified (Supporting Information). Thirty-nine glycoproteins were found commonly to have both *N*- and *O*-glycans in these data sets. Tissue expression enrichment analysis revealed that these 39 proteins and the other identified *O*-glycoproteins are highly enriched in the brain (*P* = 1.5 × 10^–9^ and 3.6 × 10^–12^, respectively) and were involved in similar processes in the brain as described in the next section for all *O*-glycoproteins identified. We performed another experiment where the same mouse brain tissue was used for both *N*- and *O*-glycoproteomics analyses by multi-lectin enrichment and the current approach, respectively (Figure S6A).^48^ While over 400 *N*-glycoproteins and over 100 *O*-glycoproteins were identified, only 24 were identified as having both *N*- and *O*-glycans (Figure S6B, Supporting Information). Of special interest, we also compared Tn glycoproteins and sites from the large-scale experiment with *O*-GlcNAcylated proteins and sites from mouse brains in previous studies.^50−53^ The overlap remained minimal (Figure S7).

MS2 spectra acquired through the HCD-pd-EThcD method identified the localization of *O*-glycosylation sites in glycoproteins, including those with sialylated *O*-glycans and the Tn antigen (Figure 2B). In these example spectra, peptide S(304)QKPGVGPGTGQPTNR with HexNAc(1)Hex(1)NeuAc(2) at S304 was identified from chondroitin sulfate proteoglycan 5 (Cspg5), while peptide T(236)CRPDEFQCADGSCIHGSR with the Tn antigen at T236 was identified from the low-density lipoprotein receptor (Ldlr). Diagnostic oxonium ions were observed from these spectra, including *m*/*z* 274 and *m*/*z* 292 for sialic acids. Both HCD spectra triggered EThcD fragmentation, resulting in the confident localization of the glycans. With other approaches published previously, the former peptide would be detected with a HexNAc(1)Hex(1) or HexNAc(1) glycan, while the latter might not be detected.

### Brain *O*-Glycoproteins Are Commonly Modified with Sialylated Core 1 *O*-Glycans

**Figure 3:**
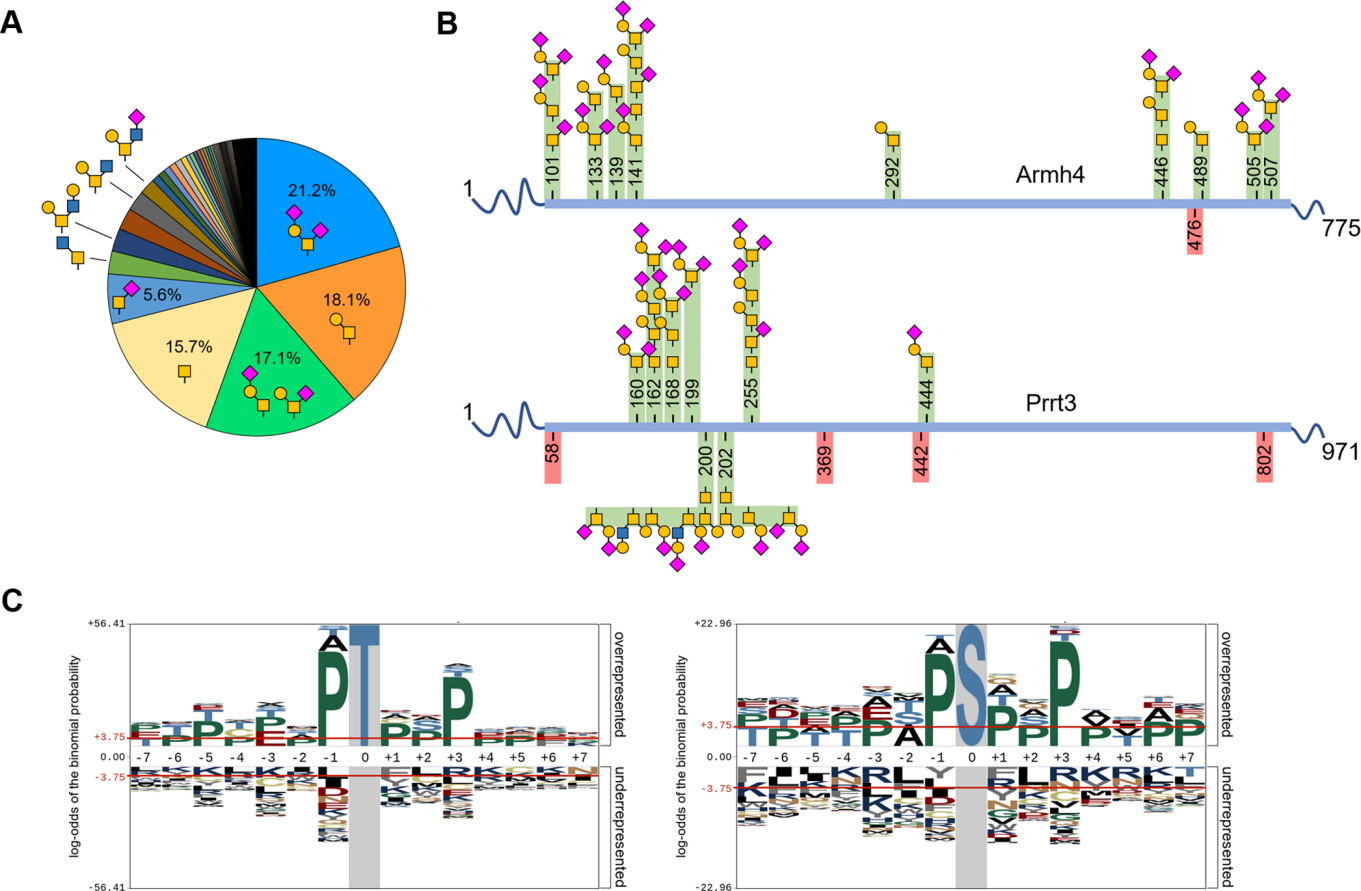
*O*-glycans identified from mouse brain glycoproteins. (A) Frequency of *O*-glycans in mouse brains. Possible structures of some high-frequency glycans are shown. (B) Examples of glycoproteins with identified *O*-glycans. All glycosylation sites were predicted by NetOGlyc to be *O*-glycosylated. Armh4 was predicted to have an *N*-glycosylation site (in pink) but has not been in identified the three data sets. In contrast, *N*- and *O*-glycosylation on Prrt3 were identified experimentally. See the full list in the Supporting Information. (C) Sequence logo of *O*-glycosylation sites identified from mouse brains generated using pLogo. Serine or threonine was centered at a time for each logo. See the full logo in Figure S11.

With site-specific glycan localization achieved by a combination of IMPa and EThcD fragmentation, we profiled the glycosylation on individual glycosylation sites, especially sialylated *O*-glycans and the Tn antigen, on a large scale. The most abundant *O*-glycan in the brain has the HexNAc(1)Hex(1)NeuAc(2) composition, which is assigned as the core 1 *O*-glycan with two sialic acid residues (Figure 3A). Other identified *O*-glycans were predicted to be core 1 *O*-glycan, mono-sialylated core 1 *O*-glycan, Tn antigen, sTn antigen, and several other structures (Figure 3A). The identification of the Tn antigen in several wild-type mouse brain glycoproteins may indicate the existence of the Tn antigen in normal tissues at basal levels, not readily detectable by antibody or lectin reagents. We also compared three search engines including pGlyco3, Byonic, and Proteome Discoverer and confirmed that the Tn antigen was still identified in normal mouse brains (Supporting Information). Note that we also compared O-Pair, MSFragger-Glyco, and GlycReSoft, but the software crashed after one day, could not localize more than one O-site, or could not perform a non-specific search, respectively. The low-level expression of the Tn antigen at specific sites and some glycoproteins may be due to reduced acceptor activity toward the T-synthase (C1GalT1), which is the only enzyme capable of adding galactose to the Tn antigen.^54^ Our results are consistent with prior studies reporting the Tn antigen on select glycoproteins expressing the Tn antigen in the normal mouse brain.^55^ Our previous experiments showed that when Tn-negative cells are examined with ReBaGs6,^56^ a specific antibody for the Tn antigen, no surface staining by this antibody was observed. However, a subtle staining was observed in the Golgi apparatus of some cells, which could arise from Tn-positive precursor glycoproteins that could be further modified by other glycosyltransferases. In recent studies using other methods, we investigated the *N*- and *O*-glycomes of mouse and human brains.^57^ However, we did not observe the Tn antigen by both VVA staining and MALDI analysis, but this could indicate the higher sensitivity of the approach used here. Overall, our glycomics analyses revealed that the large portion of brain *O*-glycans are di-sialylated core 1 *O*-glycans. In prior glycomics studies, *O*-mannosylation was also observed at a high frequency, in contrast to the results here. This is possibly due to the specificity of the IMPa, which likely does not cleave glycopeptides with *O*-mannosylation.

The majority of the glycosylation sites were threonine (67.1% for T and 32.9% for S). On average, we detected 2.4 glycosylation sites per glycoprotein, with 61% of the glycoproteins detected with only 1 glycosylation site. Two examples of *O*-glycoproteins that were identified are armadillo-like helical domain-containing protein 4 (Armh4) and proline-rich transmembrane protein 3 (Prrt3) (Figure 3B). Both glycoproteins were predicted to be *N*-glycosylated by NetNGlyc.^49^ However, *N*-glycosylation on Armh4 was not identified experimentally in the three data sets in the previous section.^17,22,48^ On the contrary, both *N*- and *O*-glycosylation on Prrt3 were identified experimentally. Of special interest is the versican core protein (Vcan), which we identified to have over 60 *O*-glycosylation sites and more than 200 glycoforms, the highest overall (Figure S8).

In another example, we also identified the glycosylation profile of the neurocan core protein (Ncan). This chondroitin sulfate proteoglycan contains 1268 amino acid residues and plays an important role in axon guidance and neurite growth.^58^*O*-glycosylation was only detected from residues 471–870 of the protein (all disordered regions) (Figure S9). The protein was heavily sialylated with most sites containing core 1 *O*-glycan and its sialylated forms and the Tn antigen. Some glycosylation sites in Ncan only have one glycan type, such as the core 1 structure at S707, while others, such as site T864, were identified with 21 different glycan compositions. This also demonstrated the complexity of protein *O*-glycosylation and the ability of this new approach to decipher the glycosylation profiles. The murine glycosyltransferase transcriptome using single-cell RNA-seq is also depicted in Figure S10, showing that glycan and glycoprotein expression within the brain may vary by region and cell type.

In contrast, N-glycosylation generally requires the consensus motif N-X-S/T, where X can be any amino acid residues except proline, and there were no clear motifs associated with *O*-glycosylation sites. We performed a sequence logo analysis of the identified glycosylation sites using pLogo.^59^ The glycosylation sites were positioned at the center of the peptide, and the sites were extended 7 residues in both directions. The mouse proteome was used as a background. As expected, both serine and threonine are significantly enriched at the 0 position of the logo, with a higher frequency of threonine over serine (Figures 3C, S11). Proline residues were also enriched in all positions from −7 to +7, especially at the −1 and +3 positions, as well as serine and threonine at the −2, +2, and +3 positions. This has also been observed in the EXoO method, which targets mainly core 1 *O*-glycoproteins.^29^ While this P-S/T-XX-P may indicate the higher chance of *O*-glycosylation, it might be affected by an unknown or cryptic specificity of IMPa. Riley and Bertozzi recently investigated *O*-glycoprotease substrate preference using five glycoprotein standards and also showed that IMPa had some preferences for alanine and proline at the −1 position.^60^ Vainauskas and colleagues showed that IMPa digests glycosylated peptides with various amino acid residues at the −1 position, except for arginine (38%), isoleucine (8%), and aspartic acid (no digestion).^31^ We observed the underrepresentation of lysine and arginine, as well as leucine, isoleucine, aspartic acid, glutamic acid, tyrosine, and cysteine in the vicinity of the *O*-glycosylation site as well (Supporting Information). They also showed that IMPa does not cleave between two adjacent *O*-glycosylation sites. Further studies will be done in the future using different cell types and tissue samples to reveal more information about protein *O*-glycosylation.

## Conclusions

Here, we modified the EXoO method for *O*-glycoproteomics analysis by including IMPa for *O*-glycopeptide cleavage and employing EThcD for site-specific analysis. This approach overcomes limitations in previously reported methods by allowing the detection of various *O*-glycopeptides including those sialylated. We demonstrated two approaches, one for total *O*-glycoproteomics analysis and the other for Tn-focused experiments by employing different methods to pre-enrich glycopeptides. We were successful in using these approaches to identify hundreds of different glycoproteins in the *O*-glycoproteome of the mouse brain. With high-pH 2D fractionation, we also improved the coverage by ∼3 times. We discovered that mouse brain *O*-glycoproteins are mostly modified with sialylated core 1 *O*-glycoproteins, which would have been ambiguously defined, at best, as core 1 *O*-glycan or the Tn antigen by other previously reported methods. Furthermore, we discovered the presence of Tn-containing glycoproteins in the mouse brain, albeit at relatively low abundance. This modification is known to be associated with disorders and not normally expressed in normal tissues.^43^ Also of interest is the largely non-overlapping identification of glycoproteins with *O*-glycans (*O*-glycoproteome) compared to those with *N*-glycans (*N*-glycoproteome). Whether these features arise from exclusivity in terms of differences in methods of analysis, *N*- versus *O*-glycosylation pathways or some other biosynthetic uniqueness to these pathways dependent on the proteins or other factors is intriguing but remains to be explored. Of special interest, a comparison of enzymatic cleavages by OgpA and IMPa, and newer *O*-glycoproteases that have been recently discovered such as SmE,^34^ on a large scale would also reveal the substrate preference for these *O*-glycoproteases. The limiting factor that may arise is the large protein amount required in our method. In summary, the *O*-glycoproteomic information and approaches presented here present important new information and a new direction for exploring *O*-glycosylation and functions in the brain and other organs.

## Data Availability

Raw files can be accessed through ProteomeXchange with identifier PXD037415.^61^

## Supporting Information Available

Additional experimental details, materials, and methods; scheme for large-scale identification of mouse brain *O*-glycoproteins; 138/144 ratio distribution; gene ontology enrichment and clustering of mouse brain *O*-glycoproteins; comparison of brain *N*- and *O*-glycoproteins; identification of *N*- and *O*-glycoproteins from the same mouse brain; comparison of *O*-GlcNAcylated and *O*-GalNAcylated proteins; *O*-glycosylation profiles of Vcan and Ncan; brain glycosyltransferase expression (PDF)
Supporting Table of *O*-glycopeptide Identification (xlsx)
