# Supplementary material for: Large-Scale and Site-Specific Mapping of the Murine Brain O-Glycoproteome with IMPa
Source: Anal Chem. 2023 Aug 25;95(36):13423–30. doi: 10.1021/acs.analchem.3c00408 (PMC10501376; doi:10.1021/acs.analchem.3c00408)
Supplement: Supplementary file 1 — ac3c00408_si_001.pdf [file ac3c00408_si_001.pdf]

Supporting Information

Large-scale and Site-specific Mapping of the Murine Brain O-Glycoproteome with IMPa

Suttipong Suttapitugsakul[1], Yasuyuki Matsumoto[1^], Rajindra P. Aryal[1], and Richard D. Cummings[1,*]

[1]Department of Surgery, Beth Israel Deaconess Medical Center, Harvard Medical School, Boston, MA, 02215, USA

[^]Current Address: Office of Biotechnology Products, Center for Drug Evaluation and Research, Food and Drug Administration, Silver Spring, MD

***Corresponding author:**

Richard D. Cummings - National Center for Functional Glycomics, Department of Surgery, Beth Israel Deaconess Medical Center, Harvard Medical School, CLS 11087 - 3 Blackfan Circle, Boston, MA, 02215, USA. Tel: 1-617-735-4643, rcummin1@bidmc.harvard.edu, ORCID- 0000-0002-8918-5034

Table of Contents

Experimental Section

Supplementary Figures S1-S11 and Figure Legends

Experimental Section

Cell culture and tissue samples

Reagents were obtained from Sigma-Aldrich unless noted otherwise. Human embryonic kidney (HEK293) cell line and the corresponding Tn^+ cells (SimpleCell), which were generated by deletion of the *Cosmc* gene, were a kind gift from Dr. Henrik Clausen (University of Copenhagen).[1] HEK293 cells were cultured in Dulbecco's Modified Eagle's Medium (DMEM, Corning) supplemented with 10% (vol/vol) fetal bovine serum (Atlanta Biologicals) and 200 units/mL penicillin-streptomycin (Thermo) at 37°C and 5% CO_2. Cultured HEK293 cells were rinsed 3x with PBS (Corning), incubated with 0.02% EDTA in PBS for 5 min at RT, and collected after centrifugation at 400*g* for 5 min at 4°C. The cell pellets were rinsed 2x with ice-cold PBS and stored at -80°C before use.

Fresh postmortem mouse brains were collected from wild-type C57BL/6J mice (Jackson Laboratory). The mice were male and aged up to ~2 years old. The mice were euthanized with CO_2, followed by cervical dislocation and immediate decapitation. The whole mouse brain was immediately removed, rinsed with ice-cold PBS, and frozen using dry ice. The brain tissues were stored at -80°C before use. All mice colonies were maintained in accordance with the guidelines established by the Animal Care and Use Committee at Beth Israel Deaconess Medical Center.

Cell lysis, protein extraction, and peptide purification

HEK293 cells were lysed in an ice-cold lysis buffer containing 50 mM HEPES pH=8.6, 0.5% sodium deoxycholate, 150 mM sodium chloride, and cOmplete protease inhibitor cocktail EDTA-free (Roche). The cells were sonicated three times with a Qsonica sonicator for 10 s each at 20 A with 30 s break between rounds, followed by incubation at 4°C with end-over-end rotation for 45 min. Brain tissues were homogenized with a Dounce homogenizer on ice before the sonication. The lysates from both HEK cells and mouse brain tissues were centrifuged at 3220*g* for 10 min and the debris were discarded. The protein concentration was measured with BCA assay. The proteins were reduced with 5 mM dithiothreitol at 56°C, 30 min and subsequently alkylated with 14 mM iodoacetamide at room temperature in the dark for 30 min.[2] The proteins were purified with the methanol/chloroform precipitation method. The pellets were dried and resuspended in a digestion buffer containing 50 mM HEPES pH=8.6, 1.6 M urea, and 5% acetonitrile (ACN). The proteins were digested with sequencing grade modified trypsin (Promega) at the protein:enzyme ratio of 100:1 at 37°C with shaking overnight.

Enzymatic release of O-glycopeptides with IMPa

Methods for glycopeptide extraction and immobilization were adapted and modified from Yang *et al.*[3-4] After protein digestion, the mixture was acidified with trifluoroacetic acid (TFA) to pH<2, clarified by centrifugation, and desalted with C18 Sep-Pak Vac cartridges (Waters). Before the elution step, the columns were also loaded with a solution containing 0.95 M ammonium hydroxide, 0.2 M O-methylisourea hemisulfate salt, and 0.03% TFA, and incubated at 65°C for 20 min to convert lysine into homoarginine. This prevents lysine from being conjugated to AminoLink beads during the reductive amination reaction and thus the glycopeptides can be released after IMPa cleavage. The eluted peptides were dried by lyophilization. For total O-glycoprotein experiments, the peptides were resuspended in 95% ACN with 1% TFA. The glycopeptides were enriched with HyperSep™ Retain AX Cartridges (RAX, Thermo). RAX functions similar to SAX-ERLIC.[5-7] However, the cartridge consists of a polystyrene-divinylbenzene polymer functionalized with a quaternary amine group. Similar to previous studies[3-4, 8], RAX cartridges were conditioned with 1) ACN, 2) 100 mM pH=7 triethylammonium acetate, 3) deionized water, and 4) 95% ACN/1% TFA. The peptides were then loaded and washed with 95% ACN/1% TFA. Eventually, enriched glycopeptides were eluted with 50% ACN/0.1% TFA. For Tn-focused experiments, the peptides were resuspended in PBS and enriched with agarose-bound VVA (Vector) overnight at room temperature according to the manufacturer's protocol. The glycopeptides were eluted by incubating with a solution containing 4 M urea, 100 mM Tris-HCl pH=7.4, and 400 mM N-acetyl-D-galactosamine (Carbosynth) for 30 min at room temperature with shaking. The eluted glycopeptides were desalted with C18 Sep-Pak Vac cartridges. Glycopeptides from both approaches were dried via lyophilization.

The resulting glycopeptides were resuspended in PBS and the pH was adjusted to ~7. The peptide amount was determined with a nanodrop spectrophotometer. The peptides were conjugated to AminoLink™ Plus Coupling Resin (Thermo) at the peptide:bead ratio of 1 μg:1 μL in the presence of 50 mM sodium cyanoborohydride with end-over-end rotation at room temperature overnight. The beads were subsequently blocked by a reaction with 1 M Tris-HCl pH=7.5 with 50 mM sodium cyanoborohydride for 30 min at room temperature with end-ever-end rotation. The beads were washed four times with 1) 60% ACN and 0.1% TFA, 2) 1.5 M sodium chloride, and 3) 20 mM Tris-HCl pH=7.5. The beads were resuspended in 20 mM Tris-HCl pH=7.5. O-glycopeptides were released by IMPa O-glycoprotease (New England BioLabs) at the peptide:enzyme ratio of 10 μg:1 μL. The reaction took place at 37°C with end-over-end rotation overnight. Next day, the supernatant was collected. The beads were washed twice with 0.1% TFA and pooled with the supernatant. The pooled eluate was desalted with a C18 Sep-Pak cartridge and dried with lyophilization.

High-pH peptide fractionation

For the large-scale mouse brain experiment, the dried glycopeptides were fractionated with high-pH reversed-phase using a Shimadzu 20A HPLC system. Peptides were fractionated using an XBridge C18 3.5 μm, 4.6x250 mm column (Waters). Buffer A was 10 mM ammonium formate pH=10 in water and buffer B was 10 mM ammonium formate pH=10 in 90% ACN. The peptides were fractionated and collected during a 50-minute gradient of 5% to 35% buffer B and the flow rate of 0.7 mL/minute. The peptides were collected every 2 minutes, consolidated into 12 fractions, and dried with lyophilization. The peptides were desalted with a C18 Sep-Pak cartridge and dried with lyophilization.

LC-MS/MS analysis

Peptide sequencing was performed on a Dionex UltiMate 3000 UHPLC system coupled to an Orbitrap Fusion Lumos Tribrid mass spectrometer (Thermo). The glycopeptides were dissolved in a solution containing 0.1% formic acid (FA) in water and loaded onto a C18 precolumn (C18 PepMap 100, 300 μm × 5 mm, 5 μm, 100 Å, Thermo) with 15 μL/min solvent A (0.1% FA in water) for 3 min, and separated on a C18 analytical column (PicoFrit 75 μm ID × 150 mm, 3 μm, New Objective) using a gradient of 2-40% solvent B (80% ACN and 0.1% FA in water) over 95 min, followed by a gradient of 40-90% over 3 min. The ion source voltage was 2100 V. The ion transfer tube temperature was 275°C. Higher-energy dissociation product ions-triggered electron-transfer/higher-energy collision dissociation (HCD-pd-EThcD MS2) or HCD MS2 alone was used for glycopeptide identification. With the HCD-pd-EThcD approach, the mass spectrometer was operated in a data-dependent acquisition mode where 15 most intense ions from MS1 were sequenced in MS2 with HCD, and those with specified oxonium ions in MS2 were also sequenced with EThcD at MS2 level. MS1 was performed in the Orbitrap at the resolution of 120,000. Depending on the experiment, the MS1 scan range was set to m/z 350-2000 or m/z 800-2000. Normalized AGC target was set 100% with the maximum ion injection time of 50 ms. The quadrupole was used to isolate precursor ions with the isolation window of 1.6 m/z. Dynamic exclusion was employed for 10 s with the mass tolerance of 10 ppm after 1 time. Stepped HCD with collision energies of 20%, 30%, and 40% was used to fragment the peptides in HCD MS2. MS2 detection was performed in the Orbitrap with the resolution of 30,000 and the first m/z of 100. The normalized AGC target was set to 100% with the maximum injection time of 250 ms. Targeted mass trigger was used after HCD MS2 where at least two ions with m/z=204.0865, 168.0654, 186.0760, 274.0921, 292.1027, 366.1395, 126.0550, 138.0549, or 144.0655, with mass tolerance of 25 ppm and were one of the 20 most intense ions triggered another MS2 with EThcD.[9] Calibrated charge-dependent ETD was enables with ETD supplemental energy of 25%. Ions were detected in the Orbitrap with a 30,000 resolution in the high mass range mode with the scan range from m/z=120-4000. The normalized AGC target was 200% and the maximum ion injection time was 200 ms. For test run experiments with HCD MS2, the mass spectrometer was operated in a data-dependent acquisition mode where 15 most intense ions from MS1 were sequenced in MS2 with HCD. MS1 was performed in the Orbitrap at the resolution of 60,000. MS1 scan range was set to m/z 400-1600. Normalized AGC target was set to 25% with the maximum ion injection time of 50 ms. The quadrupole was used to isolate precursor ions with the isolation window of 1.5 m/z. Dynamic exclusion was employed for 10 s with the mass tolerance of 20 ppm after 1 time. Stepped HCD with

collision energies of 25%, 30%, and 35% was used to fragment the peptides in HCD MS2. MS2 detection was performed in the Orbitrap with the resolution of 30,000 and the first m/z of 100. The normalized AGC target was set to 400% with the maximum injection time of 250 ms.

Database searching and bioinformatics analysis

Raw files were searched using pGlyco3[10] against the human proteome database (downloaded on 10/05/2022, reviewed, containing both canonical and non-canonical sequences, 42,397 entries) or the mouse (*Mus musculus*) proteome database (downloaded on 06/09/2021, reviewed, containing both canonical and non-canonical sequences, 25,368 entries) from UniProt. Trypsin was set as an enzyme with the specificity set to semi. Maximum number of missed cleavages was 2. Carbamidomethylation of cysteine (+57.0215 Da) and guanidination of lysine (+42.0218 Da) were set as static protein modifications. Oxidation of methionine (+15.9949 Da) was set as a variable protein modification. Maximum variable modification was 2. Peptide length was from 6 to 40. The glycan database used was the default "Multi-Site-O-Glycan". Precursor ion mass tolerance was 10 ppm and fragment ion mass tolerance was 20 ppm. The glycopeptide false discovery rate (FDR) was 1%. Identified glycopeptides were inspected and filtered manually. In general, we checked for the presence of c- and z- ions and the localization of the O-glycan was confirmed. Glycan composition was also validated by determining the glycan losses from the precursor ions. Other spectra were removed from the final results. Additionally, we confirmed the presence of oxonium ions at m/z=204 and, in some cases, m/z=292 and 274 for sialic acid. Only those without the N!P[S/T/C] sequon for N-linked glycosylation were retained. The 138/144 ratios were extracted with pGlycoQuant.[11] The majority of the identified and filtered GSMs have the ratio <3, indicating the high number of O-glycopeptides in the datasets. For total O-glycoproteins experiments, the identification was classified into type 1 for those with a localized site with supporting fragment ions or with a non-zero localization score. The peptides must also have a terminal serine or threonine when IMPa was used. The rest were classified as type 2. For Tn-focused experiments, type 1 were those with HexNAc(1) as the modified glycan with supporting fragment ions with a non-zero localization score, type 2 were those identified by HCD fragmentation only but modified with HexNAc(1) with a terminal serine or threonine, and type 3 were the rest. Glycosylation types can be found in the supplemental tables. Tissue enrichment analysis was performed with FUMA.[12] N-glycosylation prediction was analyzed with NetNGlyc.[13] O-glycosylation prediction was analyzed with NetOGlyc.[1] Gene-ontology clustering was performed with DAVID.[14-15] Sequence logos were generated with pLogo.[16] Figures were created with BioRender.com. OriginLab was used to create Fig. 2. We define the glycospecies as followed: glycoprotein groups, analogous to protein groups[17], refers to glycoproteins that were inferred from the glycopeptide. This could be the canonical form if it does not have any isoforms, or multiple isoforms if the identified glycopeptide cannot be used to distinguish between them, or a specific isoform if the identified glycopeptide can be used to distinguish between the isoforms. All were counted as one glycoprotein group.; glycoproteins refers to the individual glycoproteins from the glycoprotein groups. For example, if there are three possible isoforms that cannot be distinguish from one glycoprotein group, the glycoprotein count is three. Note that, it is likely that only one glycoforms was detected but cannot be distinguished among others. This might lead to an inflated number of glycoproteins reported; all glycopeptides (in the Tn glycoproteomics section) refers to all non-redundant glycopeptides with the Tn antigen or with other O-glycans; GSMs refers to glycopeptide-spectrum matches, similar to peptide-spectrum matches for non-glycosylated peptides; and glycoforms refers to the specific O-glycan on a particular glycosylation site. For example, if three O-glycans were detected on one glycosylation site, the number of glycoforms is considered as three.

N-glycoproteomics analysis

To analyze N- and O-glycoproteins from the same tissue, the proteins from mouse brain were extracted and digested similarly. About 5 mg of proteins was used for N-glycoproteomics analysis and the rest was for O-glycoproteomics analysis as described above. A combination of six agarose-bound lectins, including 250 µL Concanavalin A (ConA), 80 µL *Ricinus communis* agglutinin I (RCA), 80 µL *Sambucus nigra* agglutinin (SNA), 90 µL *Aleuria aurantia* lectin, 120 µL wheat germ agglutinin (WGA), and 80 µL *Vicia villosa* agglutinin (Vector Laboratories), were used for the enrichment. The dried and purified

peptides were dissolved in a binding buffer containing 50 mM HEPES, 1 mM $MnCl_2$, 1 mM $MgCl_2$, and 1 mM $CaCl_2$, and incubated for 1 hour at 37°C with end-over-end rotation. The beads were washed 5 times with the binding buffer. Enriched glycopeptides were eluted by incubation with 1) 200 mM methyl α-D-mannopyranoside, 200 mM methyl α-D-glucopyranoside, 200 mM D-(+)-galactose, 100 mM L-(-)-fucose, 500 mM N-acetyl-D-glucosamine, and 200 mM N-acetyl-D-galactosamine (Carbosynth), 2) 500 mM D-lactose (Fisher), and 3) 500 mM D-lactose containing 200 mM acetic acid (Chem-Impex). Each elution was incubated for 15 min with shaking at room temperature. The eluted glycopeptides were combined, desalted with a C18 Sep-Pak cartridge, and dried with lyophilization. N-glycopeptides were analyzed with LC-MS/MS using the gradient similar to the method for O-glycoproteomics analysis. MS1 was performed in the Orbitrap at the resolution of 120,000. MS1 scan range was set to m/z 800-2000. Normalized AGC target was set to 100% with the maximum ion injection time of 50 ms. The quadrupole was used to isolate precursor ions with the isolation window of 1.5 m/z. Dynamic exclusion was employed for 10 s with the mass tolerance of 20 ppm after 1 time. Stepped HCD with collision energies of 20%, 30%, and 40% was used to fragment the peptides in HCD MS2. MS2 detection was performed in the Orbitrap with the resolution of 30,000 and the first m/z of 100. The normalized AGC target was set to 100% with the maximum injection time of 250 ms. Raw files were searched using pGlyco 3. The same FASTA file from mouse was used as above. Trypsin was set as an enzyme with the specificity set to specific. Maximum number of missed cleavages was 2. Carbamidomethylation of cysteine (+57.0215 Da) was set as a static protein modification. Oxidation of methionine (+15.9949 Da) was set as a variable protein modification. Maximum variable modification was 2. Peptide length was from 6 to 40. The glycan database used was the default "pGlyco-N-Mouse". Precursor ion mass tolerance was 10 ppm and fragment ion mass tolerance was 20 ppm. The glycopeptide false discovery rate (FDR) was 1%. Identified glycopeptides were inspected and filtered manually. Only those with the N!P[S/T] sequon for N-linked glycosylation were retained.

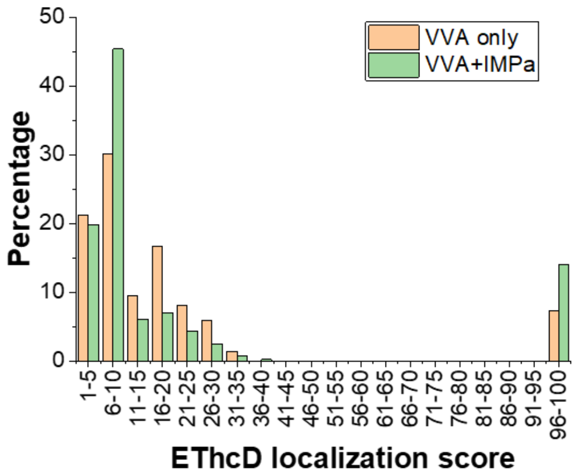

Fig. S1. Identification of Tn glycoproteins from SimpleCell HEK293 cells. Localization score of Tn glycosylation sites from VVA enrichment and from VVA enrichment combined with IMPA reaction. Both were from HCD-pd-EThcD experiments.

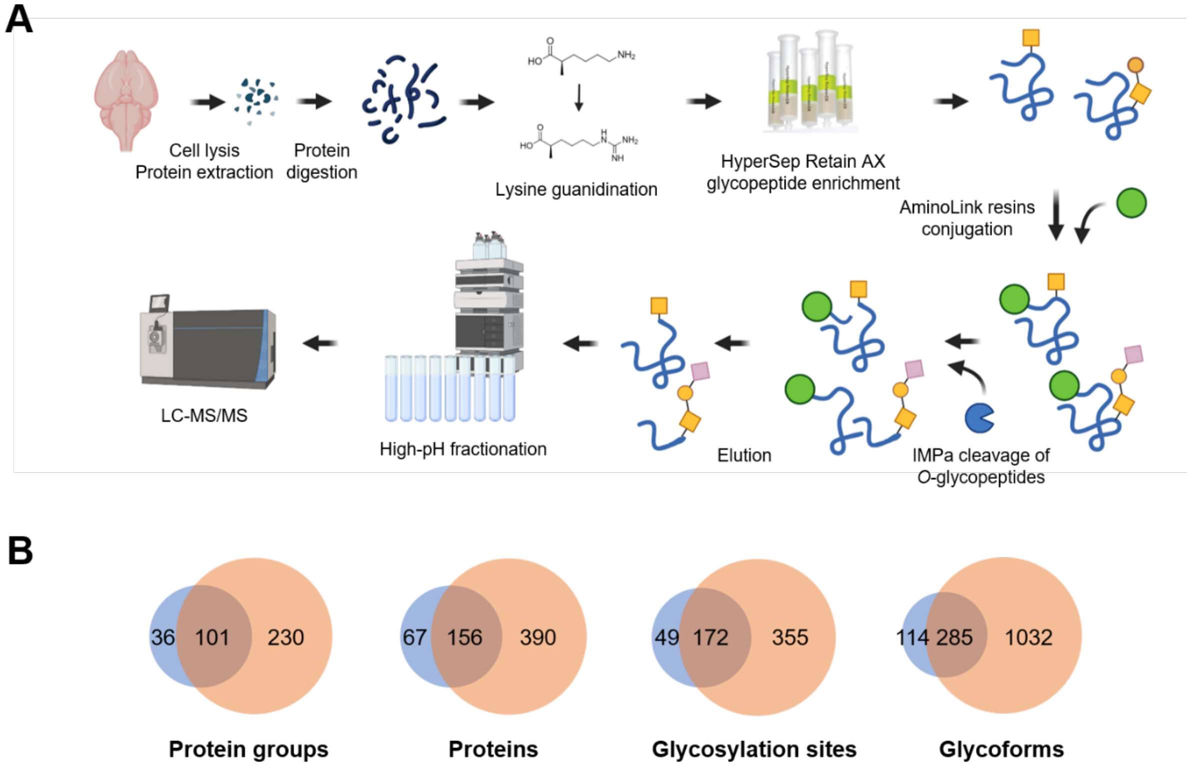

Fig. S2. Large-scale O-glycoproteomics analysis of mouse brain. (A) Workflow for the identification of O-glycoproteins in mouse brain. Here, mouse brains were processed similar to that in Fig. 1. However, after O-glycopeptides were cleaved with IMPa, high-pH reverse-phase HPLC was used to fractionate the resulting peptides into 12 fractions. Each of the fraction was analyzed with LC-MS/MS. (B) Comparison of mouse brain O-glycoproteins from single-shot experiments (from Fig. 3A, both biological duplicated were pooled) and experiments with 2D fractionation from Fig. S1A.

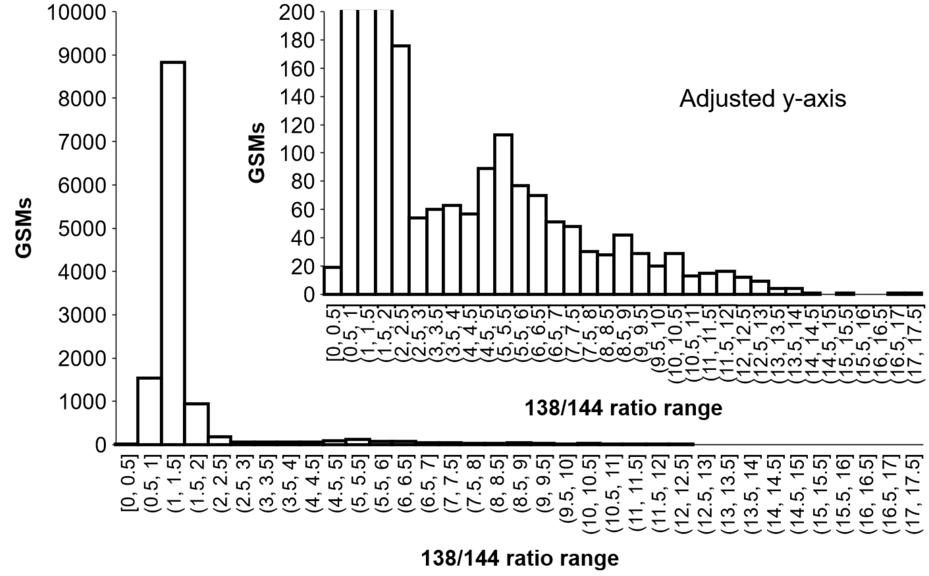

Fig. S3. 138/144 ratio of the identified O-glycopeptides from mouse brain. The intensity of m/z=138.0549 and m/z=144.0655 was extracted from MS2 spectra using pGlycoQuant with the default parameter.[11] The 138/144 ratio was calculated manually. Over 93% of the reported O-glycopeptides have the ratio lower than 3. The inset shows the same plot but the y-axis was adjusted.

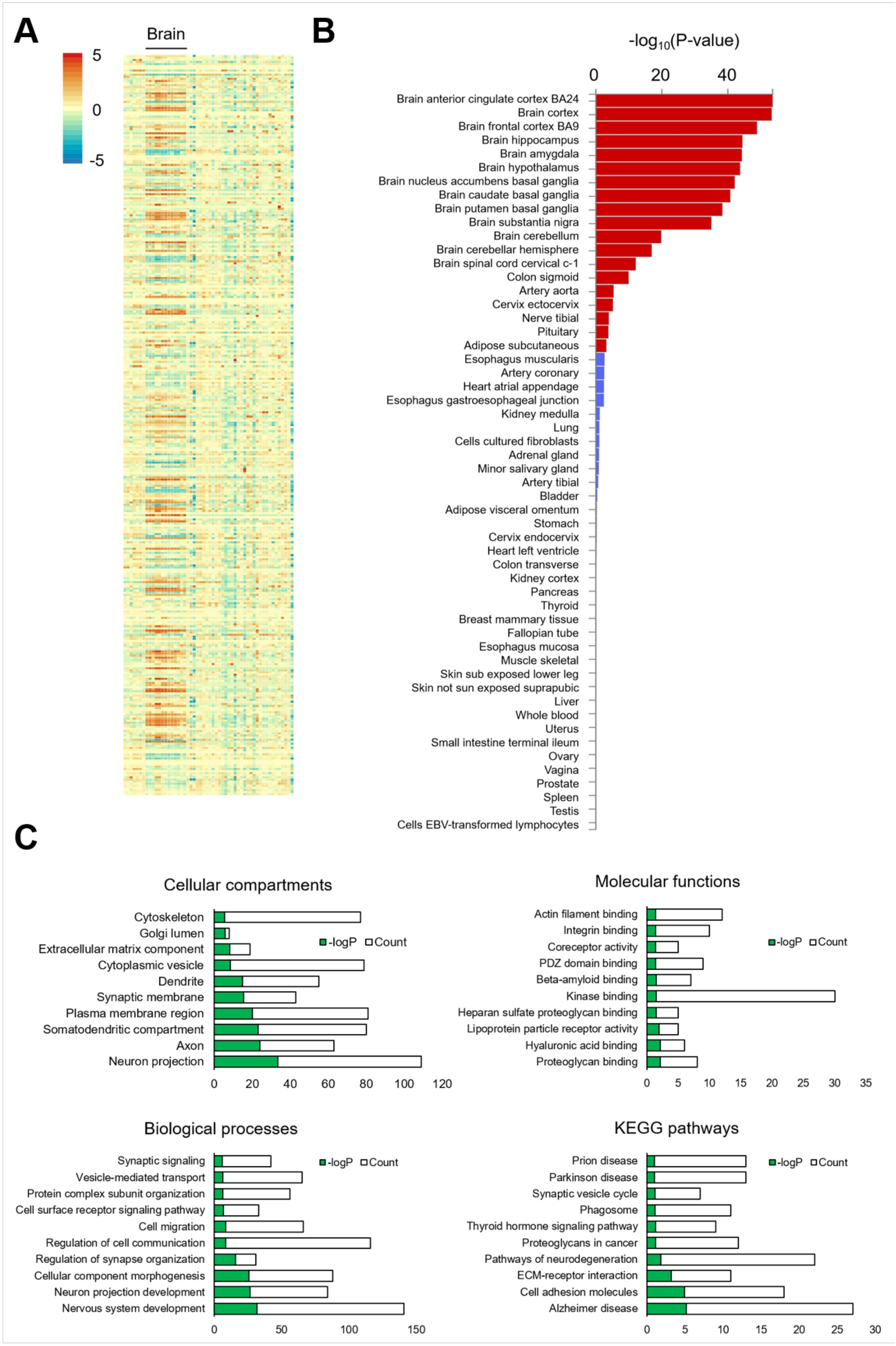

A
Brain
5
0
-5
B
-log10(P-value)
0
20
40
Brain anterior cingulate cortex BA24
Brain cortex
Brain frontal cortex BA9
Brain hippocampus
Brain amygdala
Brain hypothalamus
Brain nucleus accumbens basal ganglia
Brain caudate basal ganglia
Brain putamen basal ganglia
Brain substantia nigra
Brain cerebellum
Brain cerebellar hemisphere
Brain spinal cord cervical c-1
Colon sigmoid
Artery aorta
Cervix ectocervix
Nerve tibial
Pituitary
Adipose subcutaneous
Esophagus muscularis
Artery coronary
Heart atrial appendage
Esophagus gastroesophageal junction
Kidney medulla
Lung
Cells cultured fibroblasts
Adrenal gland
Minor salivary gland
Artery tibial
Bladder
Adipose visceral omentum
Stomach
Cervix endocervix
Heart left ventricle
Colon transverse
Kidney cortex
Pancreas
Thyroid
Breast mammary tissue
Fallopian tube
Esophagus mucosa
Muscle skeletal
Skin sub exposed lower leg
Skin not sun exposed suprapubic
Liver
Whole blood
Uterus
Small intestine terminal ileum
Ovary
Vagina
Prostate
Spleen
Testis
Cells EBV-transformed lymphocytes
C
Cellular compartments
Cytoskeleton
Golgi lumen
Extracellular matrix component
Cytoplasmic vesicle
Dendrite
Synaptic membrane
Plasma membrane region
Somatodendritic compartment
Axon
Neuron projection
-logP
Count
0
20
40
60
80
100
120
Molecular functions
Actin filament binding
Integrin binding
Coreceptor activity
PDZ domain binding
Beta-amyloid binding
Kinase binding
Heparan sulfate proteoglycan binding
Lipoprotein particle receptor activity
Hyaluronic acid binding
Proteoglycan binding
-logP
Count
0
5
10
15
20
25
30
35
Biological processes
Synaptic signaling
Vesicle-mediated transport
Protein complex subunit organization
Cell surface receptor signaling pathway
Cell migration
Regulation of cell communication
Regulation of synapse organization
Cellular component morphogenesis
Neuron projection development
Nervous system development
-logP
Count
0
50
100
150
KEGG pathways
Prion disease
Parkinson disease
Synaptic vesicle cycle
Phagosome
Thyroid hormone signaling pathway
Proteoglycans in cancer
Pathways of neurodegeneration
ECM-receptor interaction
Cell adhesion molecules
Alzheimer disease
-logP
Count
0
5
10
15
20
25
30

Fig. S4. Clustering analysis of O-glycoproteins from mouse brains. (A) Heatmap showing tissue expression of the identified glycoproteins using FUMA through GTEx v8 with 54 tissue types. The color key shows average of normalized expression per label (zero mean across samples). (B) Tissue specificity enrichment analyzed using FUMA through GTEx v8 with 54 tissue types. The tissues were ordered by up-regulated differentially expressed genes P-value. Those significantly enriched (P_{Bon}<0.05) were highlighted in red. (C) DAVID clustering based on cellular compartments, molecular functions, biological processes, and KEGG pathways of the identified glycoproteins.

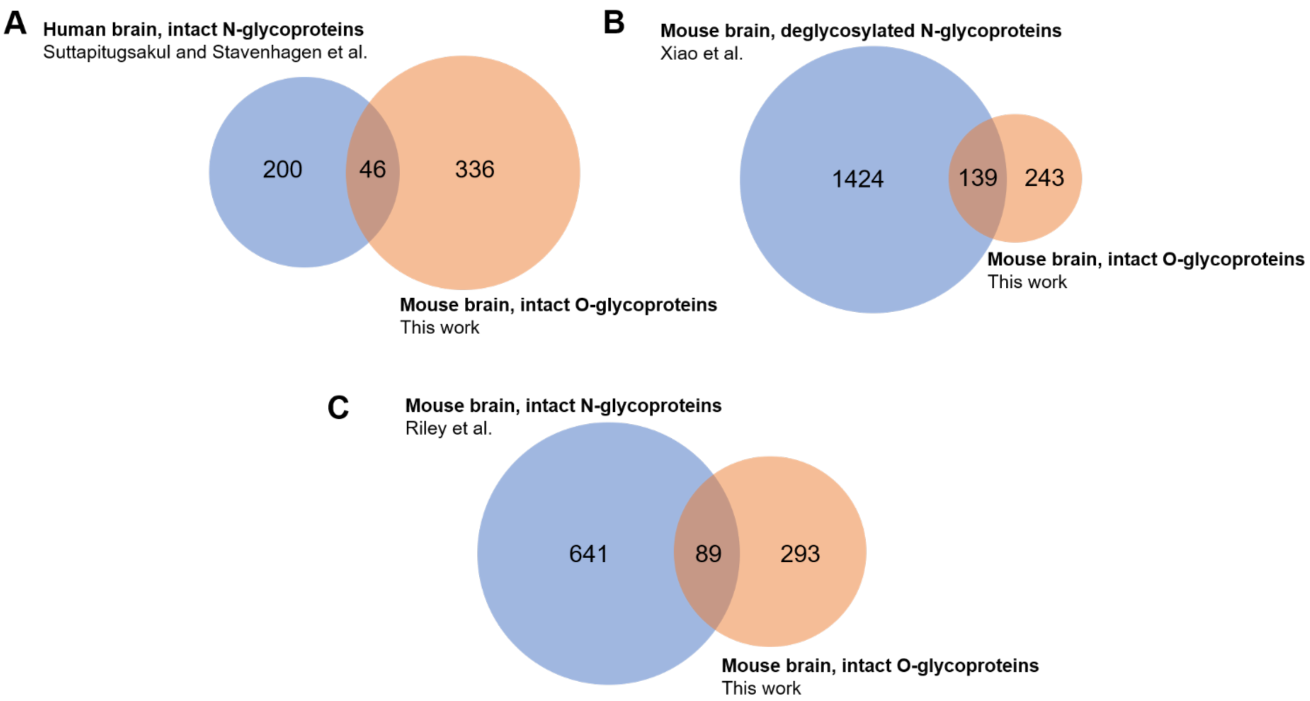

Fig. S5. Overlap of mouse brain N- and O-glycoproteins. Overlap of mouse brain O-glycoproteins with N-glycoproteins from normal human brains from intact N-glycoproteomics analysis (A), with deglycosylated N-glycoproteins from mouse brains of the same strain (B), or with intact N-glycoproteins from mouse brains of the same strains (C).

A

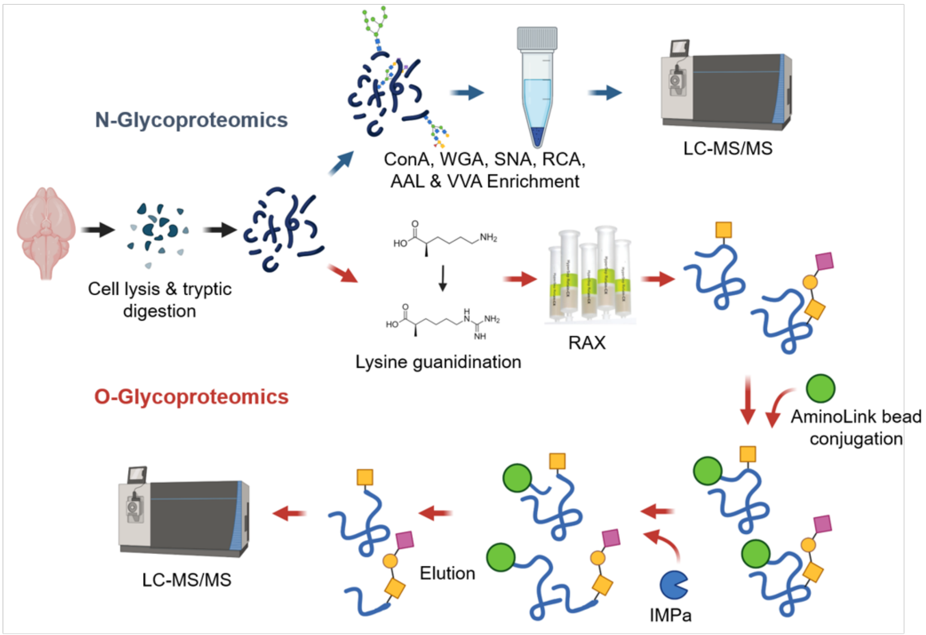

B

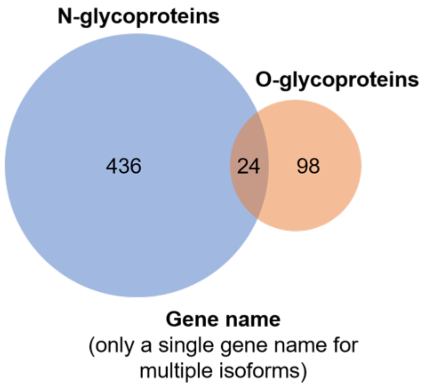

C

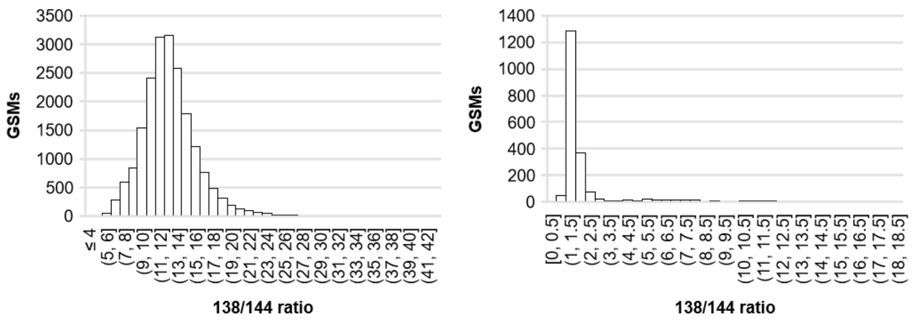

Fig. S6. Identification of N- and O-glycoproteins from the same mouse brain. (A) Experimental scheme for simultaneous identification of N- and O-glycoproteins from the same mouse brain. Mouse brain was lysed and digested into peptides. N-glycopeptides were enriched with six lectin-agarose beads while O-glycopeptides were enriched with the method shown in this work. (B) Comparison of N and O-glycoproteins identified from (A). (C) 138/144 ratio of N-glycopeptides (left) and O-glycopeptides (right).

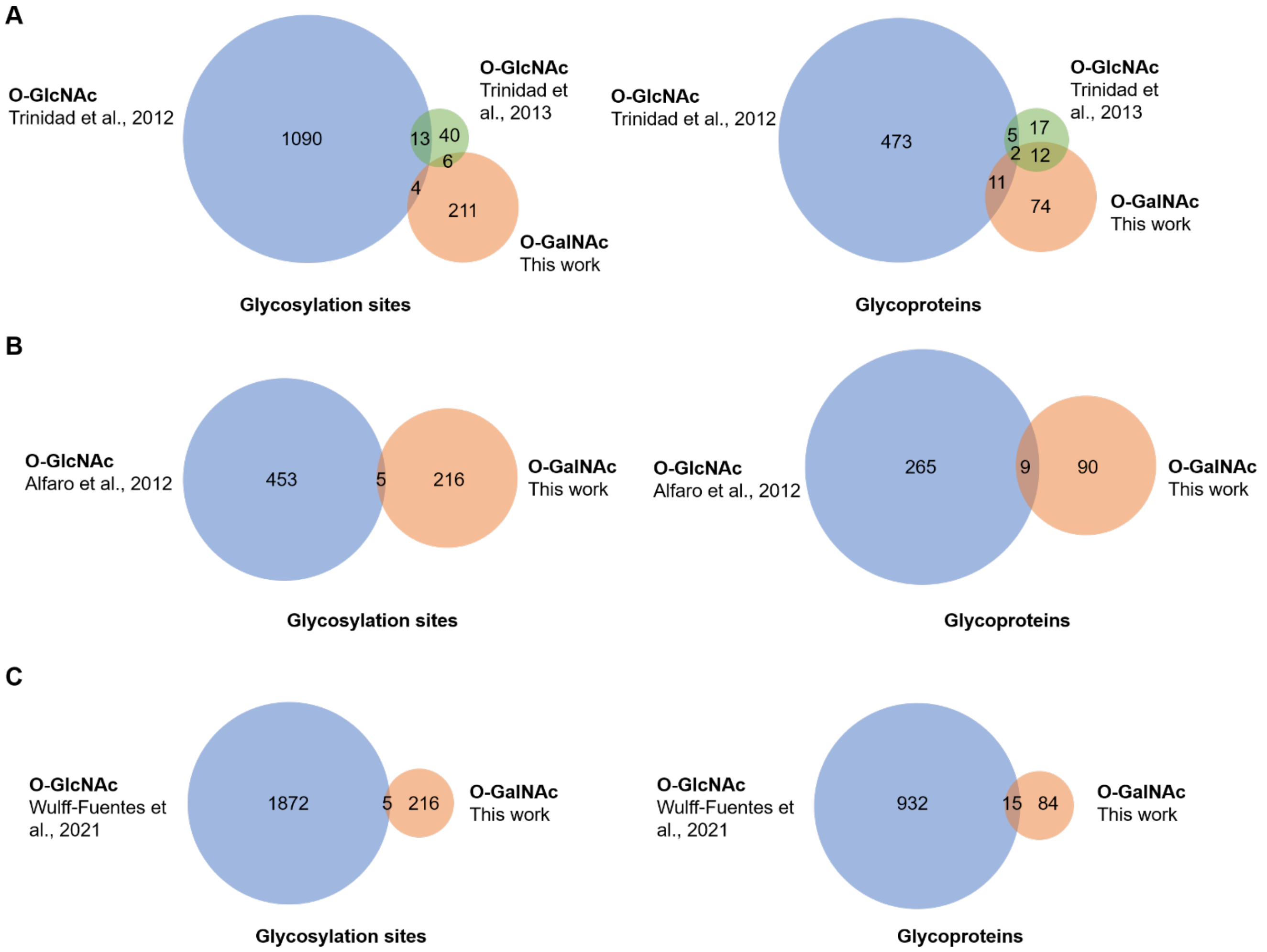

Fig. S7. Comparison of O-GlcNAcylated and O-GalNAcylated glycosylation sites and glycoproteins. (A) Comparison with O-GlcNAcylated glycosylation sites and glycoproteins from mouse synapse.[18-19] (B) Comparison with O-GlcNAcylated glycosylation sites and glycoproteins from mouse cerebrocortical brain tissue.[20] (C) Comparison with O-GlcNAcylated glycosylation sites and glycoproteins from the O-GlcNAc database.[21] Only data from *Mus musculus* were extracted.

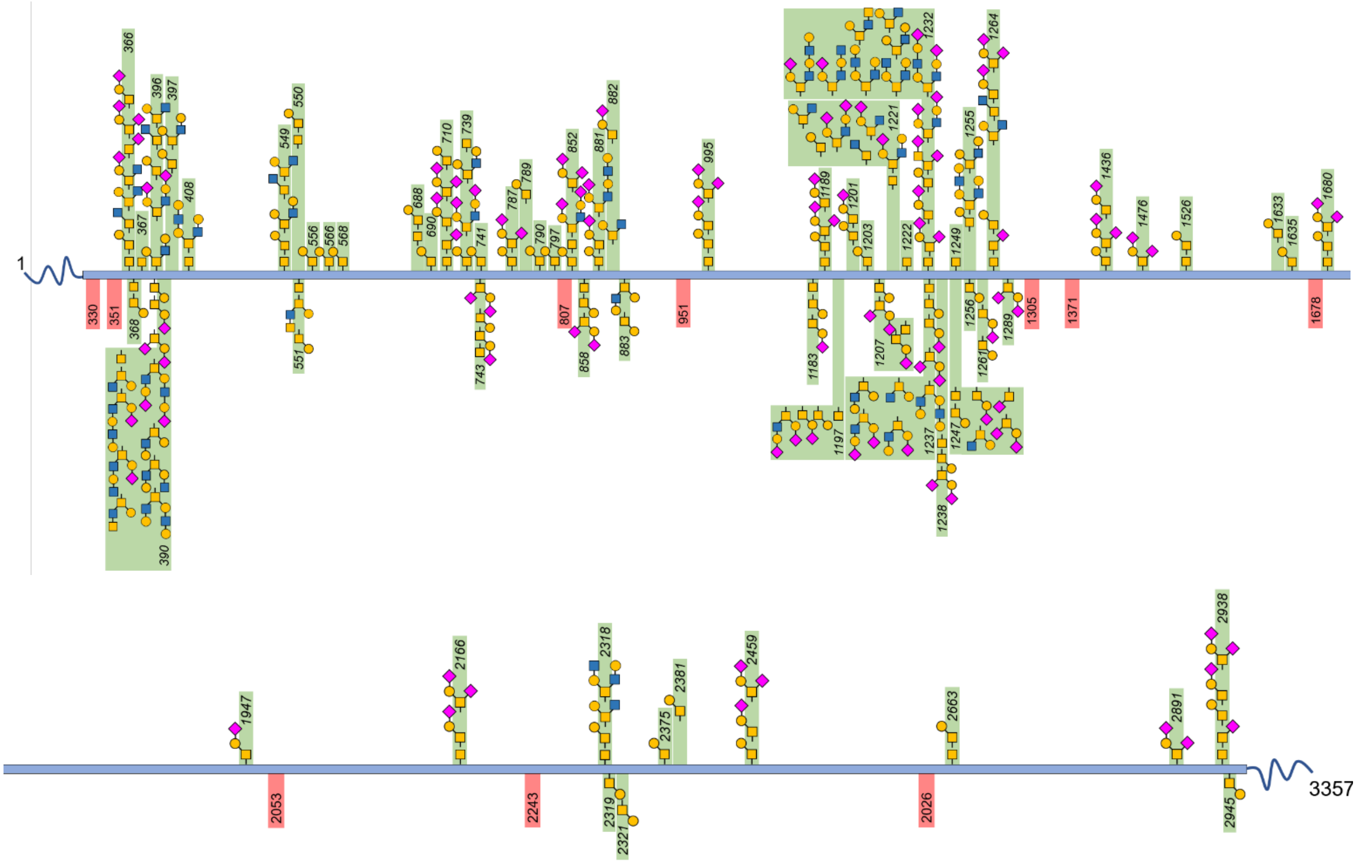

Fig. S8. O-glycosylation profile of Vcan. An example of site-specific O-glycopeptide identification. O-glycans were identified from versican core protein (Vcan). Some glycans without a predicted structure were omitted from the figure. Predicted N-glycosylation sited from NetNGlyc were shown in red.[13] Predicted O-glycosylation sites by NetOGlyc were shown in italic (all were predicted here).[1] See the full list in the Supplementary Information.

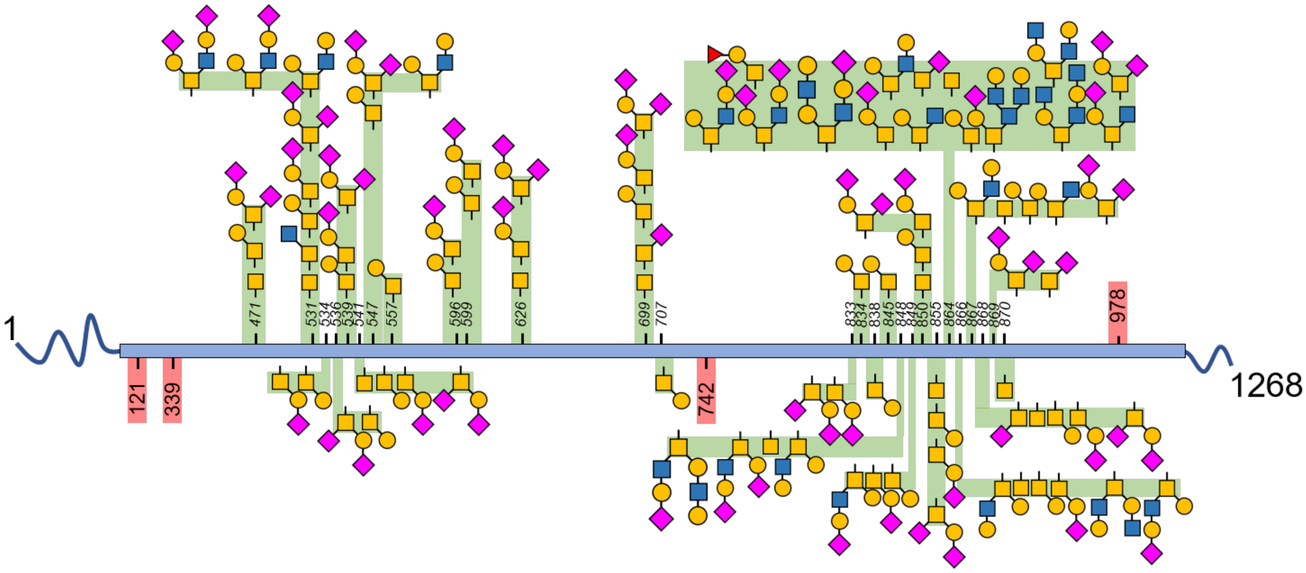

Fig. S9. O-glycosylation profile of Ncan. An example of site-specific O-glycopeptide identification. O-glycans were identified from neurocan core protein (Ncan). Some glycans without a predicted structure were omitted from the figure. Predicted N-glycosylation sited from NetNGlyc were shown in red.[13] Predicted O-glycosylation sites by NetOGlyc were shown in italic (all were predicted except for sites 838 and 850).[1] See the full list in the Supplementary Information.

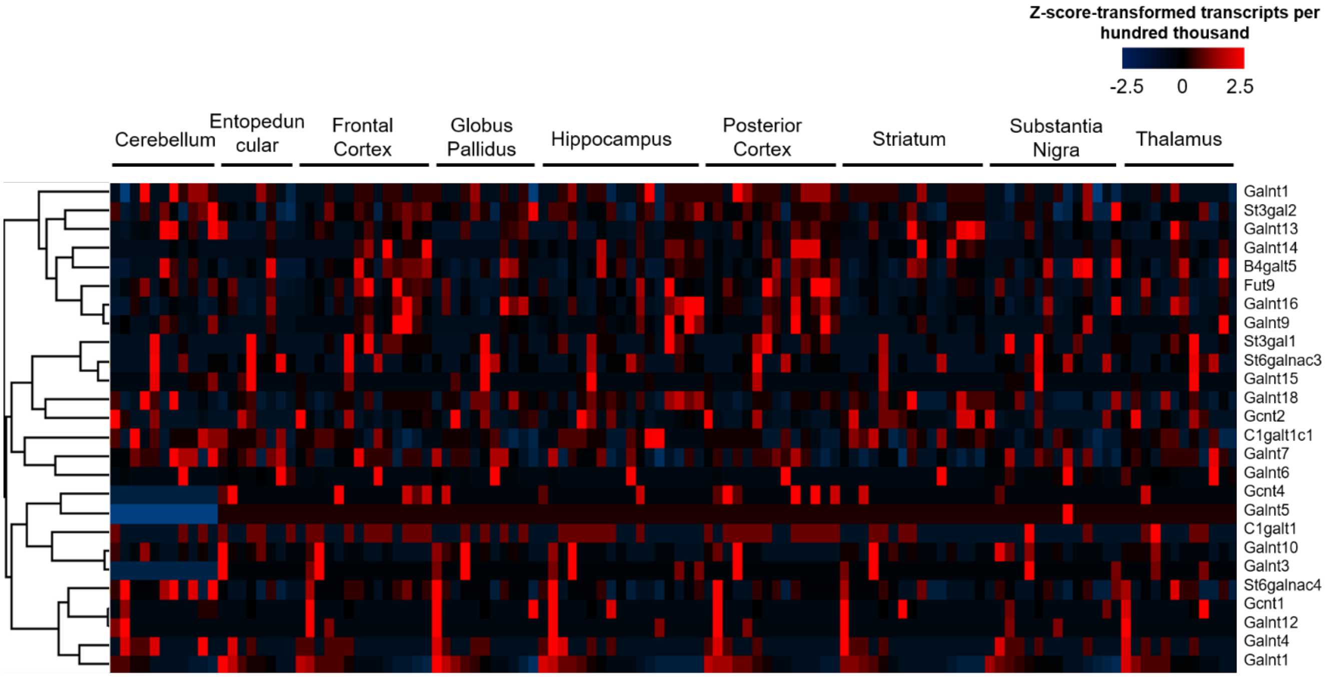

Fig. S10. RNA expression of glycosyltransferases involved in O-glycosylation. Hierarchical clustering of RNA abundance of glycosyltransferases involved in O-glycosylation from different parts of the mouse brain was extracted from DropViz.[22] Classes and clusters from the single-cell RNA-seq experiment was clustered into each brain region. Overall, the transcriptome is consistent with the types of O-glycans identified here and in our prior studies[23], and suggest that glycan and glycoprotein expression within the brain may vary by region and cell type. While here we examined the whole mouse brain, future studies on regio-specific and cell-specific O-glycoproteome and O-glycan expression, and transcriptomics within the murine brain can addressed using the broad glycoproteomic information supplied here.

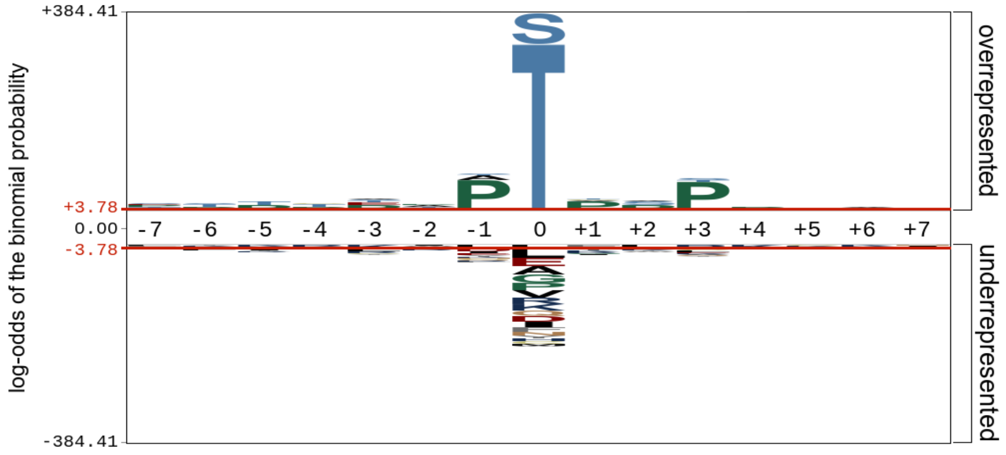

Fig. S11. Sequence logo of O-glycosylation sites. Full logo of identified O-glycosylation sites with the sites centered at the 0 position. This figure shows the overall enrichment. The full enrichment result was shown in Table S8.

References:

1. Steentoft, C.; Vakhrushev, S. Y.; Joshi, H. J.; Kong, Y.; Vester-Christensen, M. B.; Schjoldager, K. T.; Lavrsen, K.; Dabelsteen, S.; Pedersen, N. B.; Marcos-Silva, L.; Gupta, R.; Bennett, E. P.; Mandel, U.; Brunak, S.; Wandall, H. H.; Levery, S. B.; Clausen, H., Precision mapping of the human O-GalNAc glycoproteome through SimpleCell technology. *EMBO J* **2013,** *32* (10), 1478-88.
2. Suttapitugsakul, S.; Xiao, H.; Smeekens, J.; Wu, R., Evaluation and optimization of reduction and alkylation methods to maximize peptide identification with MS-based proteomics. *Mol Biosyst* **2017,** *13* (12), 2574-2582.
3. Yang, W.; Ao, M.; Hu, Y.; Li, Q. K.; Zhang, H., Mapping the O-glycoproteome using site-specific extraction of O-linked glycopeptides (EXoO). *Mol Syst Biol* **2018,** *14* (11), e8486.
4. Yang, W.; Song, A.; Ao, M.; Xu, Y.; Zhang, H., Large-scale site-specific mapping of the O-GalNAc glycoproteome. *Nat Protoc* **2020,** *15* (8), 2589-2610.
5. Alpert, A. J., Electrostatic repulsion hydrophilic interaction chromatography for isocratic separation of charged solutes and selective isolation of phosphopeptides. *Anal Chem* **2008,** *80* (1), 62-76.
6. Cao, L.; Yu, L.; Guo, Z.; Li, X.; Xue, X.; Liang, X., Application of a strong anion exchange material in electrostatic repulsion-hydrophilic interaction chromatography for selective enrichment of glycopeptides. *J Chromatogr A* **2013,** *1299*, 18-24.
7. Totten, S. M.; Feasley, C. L.; Bermudez, A.; Pitteri, S. J., Parallel Comparison of N-Linked Glycopeptide Enrichment Techniques Reveals Extensive Glycoproteomic Analysis of Plasma Enabled by SAX-ERLIC. *J Proteome Res* **2017,** *16* (3), 1249-1260.
8. Yang, W.; Shah, P.; Hu, Y.; Toghi Eshghi, S.; Sun, S.; Liu, Y.; Zhang, H., Comparison of Enrichment Methods for Intact N- and O-Linked Glycopeptides Using Strong Anion Exchange and Hydrophilic Interaction Liquid Chromatography. *Anal Chem* **2017,** *89* (21), 11193-11197.
9. Riley, N. M.; Malaker, S. A.; Driessen, M. D.; Bertozzi, C. R., Optimal Dissociation Methods Differ for N- and O-Glycopeptides. *J Proteome Res* **2020,** *19* (8), 3286-3301.
10. Zeng, W. F.; Cao, W. Q.; Liu, M. Q.; He, S. M.; Yang, P. Y., Precise, fast and comprehensive analysis of intact glycopeptides and modified glycans with pGlyco3. *Nat Methods* **2021,** *18* (12), 1515-1523.
11. Kong, S.; Gong, P.; Zeng, W. F.; Jiang, B.; Hou, X.; Zhang, Y.; Zhao, H.; Liu, M.; Yan, G.; Zhou, X.; Qiao, X.; Wu, M.; Yang, P.; Liu, C.; Cao, W., pGlycoQuant with a deep residual network for quantitative glycoproteomics at intact glycopeptide level. *Nat Commun* **2022,** *13* (1), 7539.
12. Watanabe, K.; Taskesen, E.; van Bochoven, A.; Posthuma, D., Functional mapping and annotation of genetic associations with FUMA. *Nat Commun* **2017,** *8* (1), 1826.
13. Gupta, R.; Brunak, S., Prediction of glycosylation across the human proteome and the correlation to protein function. *Pac Symp Biocomput* **2002**, 310-22.
14. Huang da, W.; Sherman, B. T.; Lempicki, R. A., Systematic and integrative analysis of large gene lists using DAVID bioinformatics resources. *Nat Protoc* **2009,** *4* (1), 44-57.
15. Sherman, B. T.; Hao, M.; Qiu, J.; Jiao, X.; Baseler, M. W.; Lane, H. C.; Imamichi, T.; Chang, W., DAVID: a web server for functional enrichment analysis and functional annotation of gene lists (2021 update). *Nucleic Acids Res* **2022,** *50* (W1), W216-21.
16. O'Shea, J. P.; Chou, M. F.; Quader, S. A.; Ryan, J. K.; Church, G. M.; Schwartz, D., pLogo: a probabilistic approach to visualizing sequence motifs. *Nat Methods* **2013,** *10* (12), 1211-2.
17. Cox, J.; Mann, M., MaxQuant enables high peptide identification rates, individualized p.p.b.-range mass accuracies and proteome-wide protein quantification. *Nat Biotechnol* **2008,** *26* (12), 1367-72.
18. Trinidad, J. C.; Barkan, D. T.; Gulledge, B. F.; Thalhammer, A.; Sali, A.; Schoepfer, R.; Burlingame, A. L., Global identification and characterization of both O-GlcNAcylation and phosphorylation at the murine synapse. *Mol Cell Proteomics* **2012,** *11* (8), 215-29.
19. Trinidad, J. C.; Schoepfer, R.; Burlingame, A. L.; Medzihradszky, K. F., N- and O-glycosylation in the murine synaptosome. *Mol Cell Proteomics* **2013,** *12* (12), 3474-88.

20. Alfaro, J. F.; Gong, C. X.; Monroe, M. E.; Aldrich, J. T.; Clauss, T. R.; Purvine, S. O.; Wang, Z.; Camp, D. G., 2nd; Shabanowitz, J.; Stanley, P.; Hart, G. W.; Hunt, D. F.; Yang, F.; Smith, R. D., Tandem mass spectrometry identifies many mouse brain O-GlcNAcylated proteins including EGF domain-specific O-GlcNAc transferase targets. *Proc Natl Acad Sci U S A* **2012,** *109* (19), 7280-5.
21. Wulff-Fuentes, E.; Berendt, R. R.; Massman, L.; Danner, L.; Malard, F.; Vora, J.; Kahsay, R.; Olivier-Van Stichelen, S., The human O-GlcNAcome database and meta-analysis. *Sci Data* **2021,** *8* (1), 25.
22. Saunders, A.; Macosko, E. Z.; Wysoker, A.; Goldman, M.; Krienen, F. M.; de Rivera, H.; Bien, E.; Baum, M.; Bortolin, L.; Wang, S.; Goeva, A.; Nemesh, J.; Kamitaki, N.; Brumbaugh, S.; Kulp, D.; McCarroll, S. A., Molecular Diversity and Specializations among the Cells of the Adult Mouse Brain. *Cell* **2018,** *174* (4), 1015-1030 e16.
23. Williams, S. E.; Noel, M.; Lehoux, S.; Cetinbas, M.; Xavier, R. J.; Sadreyev, R. I.; Scolnick, E. M.; Smoller, J. W.; Cummings, R. D.; Mealer, R. G., Mammalian brain glycoproteins exhibit diminished glycan complexity compared to other tissues. *Nat Commun* **2022,** *13* (1), 275.
